# External validation for sensitivity of the Ottawa subarachnoid hemorrhage rule in a Japanese tertiary teaching hospital

**DOI:** 10.1038/s41598-021-96320-9

**Published:** 2021-08-18

**Authors:** Tomoharu Suzuki, David Itokazu, Yasuharu Tokuda

**Affiliations:** 1 Department of Hospital Medicine, Urasoe General Hospital, 4-16-1 Iso, Urasoe-shi, Okinawa, 901-2132 Japan; 2 Okinawa Asia Clinical Investigation Synergy (www.oacis.org), Okinawa, Japan; 3 Muribushi Okinawa Project for Teaching Hospitals, Okinawa, Japan

**Keywords:** Diseases of the nervous system, Stroke, Neurology

## Abstract

The Ottawa subarachnoid hemorrhage (OSAH) rule is a validated clinical prediction rule for ruling out subarachnoid hemorrhage (SAH). Another SAH rule (Ottawa-like rule) was developed in Japan but was not well validated. We aimed to validate both rules by examining the sensitivity for ruling out SAH in Japanese patients diagnosed with SAH. We conducted a retrospective cohort study by reviewing the medical records of consecutive adult patients hospitalized with SAH at a tertiary-care teaching hospital in Japan who visited our emergency department between July 2009 and June 2019. Sensitivity and its 95% confidence interval (CI) were estimated for each rule for the diagnosis of SAH. In a total of 280 patients with SAH, 56 (20.0%) patients met the inclusion criteria and were analyzed for the OSAH rule, and a sensitivity of the OSAH rule was 56/56 (100%; 95% CI 93.6–100%). While, 126 (45%) patients met the inclusion criteria of the Ottawa-like rule, and the rule showed a sensitivity of 125/126 (99.2%; 95%CI 95.7–100%). The OSAH rule showed 100% sensitivity among our Japanese patients diagnosed with SAH. The implementation of the Ottawa-like rule should be cautious because the false-negative rate is up to 4%.

## Introduction

Subarachnoid hemorrhage (SAH) is a critical condition that may need prompt neurosurgical intervention to decrease mortality and morbidity^1^. Due to its potential fatality, making a timely diagnosis of SAH is warranted. However, while patients with acute headache account for 1% of patients who visited an emergency department, only about 0.7–2% of these patients suffer from SAH^2,3^, because of its infrequency, differentiation of SAH from other 99% causes of headache is also important in terms of radiation exposure and cost of imaging studies (e.g., computed tomography: CT). If the rule is positive, workups including CT imaging may be employed appropriately, and it may save expenditure by preventing missed SAH by the high specificity of CT imaging^4^.

Misdiagnosis of SAH was found more commonly in patients with acute headache who showed less severe symptoms^1,5^, and misdiagnosis worsened the prognosis of these patients^5^. The Ottawa subarachnoid hemorrhage (OSAH) rule is a simple clinical decision rule with high sensitivity (95% CI 97.2–100.0%) to rule out SAH in patients who visited the emergency department complaining of acute peaking headache within an hour^6^. The rule consists of six items: (1) age ≥ 40 years, (2) neck pain or stiffness, (3) witnessed loss of consciousness, (4) onset during exertion, (5) thunderclap headache (instantly peaking pain), and (6) limited neck flexion on examination. If all of the six items are absent, the patients with acute headache are thought to be at low risk of SAH.

It can be easily used for patients with acute headache who present with mild symptoms suggestive of SAH in the emergency department and contribute to rule out SAH safely. The OSAH rule has been validated as highly sensitive by studies in North America and China^7,8^.

Japan is known to have a higher incidence of SAH compared to Western and other Asian countries. The SAH incidence in Japan was estimated to be 28.0 per 100,000 person-years, and in other Asian countries, the incidence was estimated to be 3.7 per 100,000 person-years. Over the last three decades, the incidence has declined in Western and other Asian countries, but it has increased by about 60% only in Japan^9^. Thus, it is crucial to avoid a misdiagnosis of SAH, which might have different clinical manifestations in this country. On the other hand, the Ottawa-like rule (positive if any one of the following criteria is present: age ≥ 40 years, neck pain or stiffness, altered level of consciousness, or onset during exertion) was recently proposed by Japanese investigators for the assessment of patients presented emergency department complaining of an acute headache, regardless of a new neurological deficit^10^. As shown in Table 1, the OSAH rule is implemented for very specific populations, which may limit the clinical application, while the Ottawa-like rule seems to be easier to employ than the OSAH rule.

**Table 1:** The inclusion and exclusion criteria of the Ottawa subarachnoid hemorrhage rule and the Ottawa-like rule.

Inclusion criteria	The Ottawa SAH rule	The Ottawa-like rule
Age	16 years or older	No less than 15 years old
Setting	Emergency department	
Definition of headache	A nontraumatic headache that reached maximum intensity within 1 h	Acute headache
Onset of headache	Within 14 days	
Absence of a traumatic event	Previous seven days	–
Consciousness	GCS of 15/15	–
Exclusion criteria	An established recurrent headache syndrome which occurred three or more times over six months	
	An established recurrent headache syndrome which occurred three or more times over six months	
	Referred from another hospital with a confirmed diagnosis of SAH	
	Returned for a reassessment of the same headache if al- ready investigated with both CT and lumbar puncture	
	Papilledema on fundoscopic examination	
	New focal neurologic deficit	
	A previous diagnosis of a cerebral aneurysm, subarachnoid hemorrhage, brain neoplasm, or hydrocephalus	
		Headache caused by trauma, drugs or alcohol
		Unconscious at the beginning of the assessment

GCS, Glasgow Coma Scale; SAH, Subarachnoid hemorrhage.

In the current study, we aimed to assess the sensitivity of the OSAH rule and the Ottawa-like rule focusing on Japanese patients diagnosed with SAH.

## Materials and methods

### Design and setting

This was a retrospective cohort study conducted by reviewing the discharge summary of all consecutive patients who visited the emergency department and were hospitalized and eventually diagnosed with SAH at a tertiary care teaching hospital in Okinawa, Japan in a 10-year period. Data extraction was conducted by two health information managers (HIMs) according to the International Classification of Diseases, 10th Revision codes for subarachnoid hemorrhage from electronic health records (EHRs), and the data were double-checked by the HIMs. Then TS reviewed all the extracted, anonymized discharge summaries and EHRs for confirmation of diagnosis and data gathering. The diagnosis of SAH was confirmed by head CT scan, MRI, or confirmation of red blood cells (> 1 × 10^6^/L) or xanthochromia of cerebrospinal fluid. The ethics research board of the Muribushi Okinawa Project for Teaching Hospitals approved this study (No. 2019-2), and waived written informed consent due to the retrospective nature of the study.

### Cohort description

We focused on adult patients (16 years old or older) who were diagnosed with SAH from 2009 to 2019, different from a prospective validation study of the OSAH rule by Perry et al. They recruited consecutive adult patients with nontraumatic acute headache with a peak of intensity presenting within one hour from the onset. Thus, they included patients with acute headaches with or without SAH^6^.

To assess the sensitivity of the Ottawa-like rule, we excluded patients who were referred to us with a definite diagnosis of subarachnoid hemorrhage from other institutions; those who were unconscious of the initial assessment at the emergency department defined as the patient responded to pressure or not in terms of eye-opening, and their verbal responses were vague (words or sounds) or none; those who had sustained head trauma within the past one week; those who had had a similar headache three or more times in the last six months with a confirmed diagnosis or when a patient or his/her representatives opted out of participation in the study.

In addition, to assess the sensitivity of the OSAH rule, we excluded patients whose Glasgow coma scale (GCS) < 15; who had a headache for more than 14 days after onset of headache; those who had already undergone head CT and lumbar puncture and presented for reevaluation; those who had papillary edema on fundoscopy; those who presented with new focal neurological deficits; or those who had known cerebral aneurysm, subarachnoid hemorrhage, brain tumor, ventricular shunt or hydrocephalus, the same as the exclusion criteria of the validation study by Perry et al.^7^.

When one or more of item(s) of each clinical prediction rule (CPR) described in the Table 3 is applicable for the participants, they are considered as at risk of SAH.

### Data collection

Patient age, sex, past medical history, smoking history, and vital signs were obtained from the hospital chart. In addition to clinical data and demographics, data on the OSAH rule criteria were obtained from EHRs. The Ottawa-like rule (age ≥ 40 years, neck pain or stiffness, altered level of consciousness, or onset during exertion) was similarly evaluated.

### Outcome measures and statistical analyses

The outcome of interest in the current study was the sensitivity of the OSAH rule and the Ottawa-like rule in patients with SAH and its 95% confidence interval (CI).

Data were analyzed using STATA version 15 (Stata Corp LLC, College Station, TX, USA). Patients with missing data were omitted from the analysis. We reported count (proportion) for categorical variables and mean ± standard deviation (SD) for continuous variables, where appropriate.

### Consent to participate

Patients or the public were not involved in the design, conduct, reporting or dissemination of this study.

### Ethics approval

We conducted this study ethically in accordance with the World Medical Association Declaration of Helsinki. This study was an observational study without any intervention to the participants. Thus, we offered patients the opportunity to opt out by posting information about the potential eligible case for this study and the ways to contact the primary investigator.

## Results

**Figure 1:**
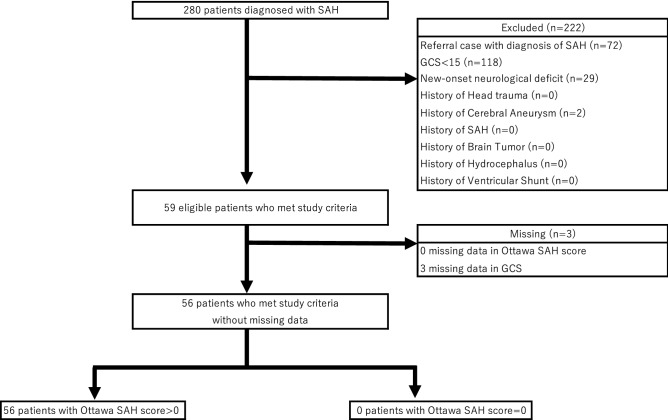
Flow diagram for the validation study of the Ottawa subarachnoid hemorrhage rule. GCS=Glasgow Coma Scale, SAH=subarachnoid hemorrhage.

We found 280 patients aged 16 years or older diagnosed with SAH during the study period. For the validation study of the OSAH rule, 222 patients were excluded. Common reasons for the exclusion were GCS < 15 (n = 118), referred cases after diagnostic confirmation of SAH at other clinics/hospitals (72), new-onset neurological deficits (29), and past history of a cerebral aneurysm (2). Three patients with missing data in the GCS or OSAH rule were also excluded. The remaining 56 patients were included in the study (Fig. 1).

**Figure 2:**
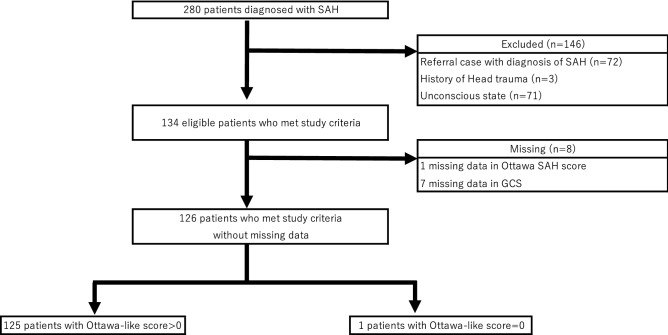
Flow diagram for the validation study of the Ottawa-like rule. GCS=Glasgow Coma Scale, SAH=subarachnoid hemorrhage.

While, we included 126 patients for the validation study of the Ottawa-like rule (Fig. 2). One hundred forty-six patients were excluded, and the reasons for exclusion were the referral case (72), patients with head trauma (3), and unconscious patients at initial presentation to our emergency department (71). Missing data were observed in GCS (7) and Ottawa-like rule (1).

Table 2 shows the demographic and clinical characteristics of the patients. Among the patients who validated the OSAH rule, the mean age was 57.5 (SD, 14.4), and there were 39 women (69.6%). Twenty-four patients had hypertension, and 20 were current or past smokers. The mean BMI was 24.2 (SD, 4.5). Median systolic blood pressure was 162 mm Hg (IQR 142–188) mm Hg. Among the patients who were included for the validation of the OSAH rule (56), head CT imaging and MR imaging found SAH in 96.4% (54/56) and 83.3% (5/6) of patients, respectively. In the patients included in the validation of the Ottawa-like rule, head CT imaging and MR imaging revealed SAH in 96.8% (122/126) and 78.6 (11/14) of the patients, respectively. Lumbar puncture was applied in two patients, and both of them presented with xanthochromia. In the cohort of the Ottawa-like rule, xanthochromia confirmed SAH in one patient with negative imaging results.

**Table 2:** Demographic characteristics of patients with subarachnoid hemorrhage.

Characteristics	The Ottawa subarachnoid hemorrhage rule (n = 56)	The Ottawa-like rule (n = 126)
Age at admission—yr	57.5 ± 14.4	57.6 ± 14.4
Female	39 (69.6)	89 (70.6)
BMI, kg/m^2^	24.2 ± 4.5	24.1 ± 4.4
**Smoking**		
Current or past smoker	20 (36.4)	48 (39.0)
**Past medical history**		
Hypertension	24 (43.6)	55 (43.7)
Dyslipidemia	3 (5.5)	7 (5.6)
**Vital signs**		
Systolic BP (mm Hg)	162 (142–188)	162 (142–189)
Diastolic BP (mm Hg)	89 (80–107)	90 (80–107)
Pulse rate (pulses/min)	83 (70–92)	82 (71–91)
Respiratory rate (breaths/min)	20 (18–22)	20 (18–24)
Body temperature (Celsius)	36.4 (35.9–36.8)	36.3 (35.9–36.7)
**Imaging study for confirmation of SAH**		
CT	54/56 (96.4)	122/126 (96.8)
MR	5/6 (83.3)	11/14 (78.6)

Reported count (proportion) for categorical variables and median (interquartile range) or mean ± standard deviation (SD) for continuous variables, where appropriate.

Lumbar puncture was applied in two patients, and both of them presented with xanthochromia. In the cohort of the Ottawa-like rule, xanthochromia confirmed SAH in one patient with negative imaging results.

Missing (Ottawa subarachnoid hemorrhage rule): Smoking history = 1; Diastolic BP = 1; Pulse rate = 9; Respiratory rate = 7; Body temperature = 15.

Missing (Ottawa-like rule): BMI = 1; smoking history = 3; diastolic BP = 1; pulse rate = 2; respiratory rate = 19; body temperature = 38.

BMI, body mass index; BP, blood pressure; CT, computed tomography; GCS, Glasgow coma scale; MR, magnetic resonance; SAH, subarachnoid hemorrhage.

As shown in Table 3, the most frequently fulfilled criterion was thunderclap headache (n = 44/46, 95.7%), followed by age ≥ 40 (n = 50/56, 89.3%). The sensitivity of the OSAH rule for the diagnosis of SAH was 56/56 (100%; 97.5% CI 95.4%–100%, one-sided). The Ottawa-like rule in our patients showed a sensitivity of 125/126 (99.2%; 95% CI 95.7%–100%). Misclassification occurred in one patient, a 30-year-old man presented with new right hemi-paresthesia. He underwent MR imaging, which showed a slight SAH; however, an emergency physician missed the finding, and the patient was discharged. Two days later, a radiologist suggested the presence of subarachnoid hemorrhage on MRI. Thus, the patient was called and admitted to our hospital immediately. Additional imaging studies did not reveal the specific etiology of SAH, such as an aneurysm or arteriovenous malformation, and he only needed close observation. Eventually, he was discharged without any disability. Because of the new neurological deficit, he was excluded from the analysis of the OSAH rule.

**Table 3:** The sensitivity of the Ottawa subarachnoid hemorrhage rule and those of the Ottawa-like rule and each criterion.

The Ottawa subarachnoid hemorrhage rule (n = 56)	%(n/N)	95% CI	The Ottawa-like rule (n = 126)	%(n/N)	95% CI
Age ≥ 40 years	89.3 (50/56)	78.1–96.0	Age ≥ 40 years	89.7 (113/126)	83.0–94.4
Neck pain or stiffness	85.7 (36/42)	71.5–94.6	Neck pain or stiffness	81.0 (68/84)	70.9–88.7
Witnessed loss of consciousness	15.1 (8/53)	6.7–27.6	–		
–			Altered level of consciousness	34.9 (44/126)	26.6–43.9
Exertional onset	62.5 (30/48)	47.4–76.0	Exertional onset	58.1 (61/105)	48.1–67.7
Thunderclap headache	95.7 (44/46)	85.2–99.5	–		
Limited neck flexion on examination	76.0 (19/25)	54.8–90.6	–		
The Ottawa SAH score ≥ 1	100 (56/56)	93.6–100*	The Ottawa-like score ≥ 1	99.2 (125/126)	95.7–100.0

Missing (OSAH rule): Neck pain or stiffness = 14; Witnessed loss of consciousness = 3; Exertional onset = 8; Thunderclap headache = 10; Limited neck flexion on examination = 31.

Missing (Ottawa-like rule): Neck pain or stiffness = 42; Exertional onset = 7.

SAH, subarachnoid haemorrhage.

*97.5% CI, one-sided.

## Discussion

Our results based on Japanese patients diagnosed with SAH show that the OSAH rule has high sensitivity, which is similar to that of the original derivation study and subsequent validation studies in North America and China^6–8,11^. Although the current study followed the study in China^8^ and was the second validation study in Asia, SAH is recognized with the highest prevalence in Japan^9^; thus, the validation is most important in this country, and physicians could use the rule to carefully exclude SAH in walk-in patients with headache safely.

The OSAH rule may be useful to rule out SAH for alert and oriented patients with acute peaking pain, although it may be implemented for a very specific population. Including patients with consciousness disturbance to be eligible for rule application, the development of another clinical decision rule for ruling out SAH was attempted in Japan by using the Ottawa-like rule with a sensitivity of 100% (95% CI 98.6–100%)^10^. Our patients showed one misclassification case and low sensitivity based on the Ottawa-like rule. The misclassified case that did not fulfil the Ottawa-like rule had a new hemi-paresthesia, and the patient was not eligible for the analysis of the OSAH rule because of the neurological deficit. The Ottawa-like rule does not exclude patients with focal neurological signs for evaluation; this may not be feasible in clinical practice. In fact, the rule missed a SAH case in our cohort; nevertheless, easier to apply than the OSAH rule.

There were several limitations in the present study that must be considered when interpreting the results. First, this study has a risk of selection bias because we included only patients with SAH in a tertiary teaching hospital and might have failed to include all patients with acute peaking headache. Thus, different from the validation study by Perry et al.^7^, we did not include all patients with acute headache without SAH. Our results focused on the sensitivity of the CPRs among patients with SAH and are at risk of overestimation and the absence of the data about patients with acute headache but not diagnosed as SAH might have weakened the validity of our results because we could not address negative predictive value or negative likelihood ratio of the OSAH rule. Second, we could apply the CPRs for a limited number of patients because of the exclusion criteria, which might have limited the clinical utility and generalizability of the CPRs. Third, this study has a possible risk for underestimation of the sensitivity of the CPRs because of the lack of data involving the CPRs. Data were retrospectively collected from EHRs in the emergency department, and physicians might have potentially failed to document some features of patients, including elements of the OSAH rule because physicians might be occasionally overwhelmed due to some reasons (ex. a severe condition of a patient with SAH, overcrowding or multitasking in an emergency department) and deprived of enough time for the documentation. Finally, this study was based on data from a single-center study with a limited number of eligible cases, so our results might have less generalizability. To overcome these limitations, a multicenter, prospective, large cohort study among patients with acute peaking headache should be planned in Japan.

## Conclusion

In summary, the OSAH rule showed 100% sensitivity among our Japanese patients diagnosed with SAH, while the Ottawa-like rule misclassified one patient with SAH. The implementation of the Ottawa-like rule should be cautious because the false-negative rate is up to 4%, and one misclassification, suggesting low generalizability when the rule is implemented for the larger Japanese population with acute headache.

## Data Availability

The datasets generated and/or analyzed during the current study are available from the corresponding author on reasonable request.

## Abbreviations

CPRs: Clinical prediction rules
GCS: Glasgow Coma Scale
OSAH: Ottawa subarachnoid haemorrhage
SAH: Subarachnoid hemorrhage
